# Joubert Syndrome in Three Children in A Family: A Case Series

**Published:** 2013

**Authors:** Javad Akhondian, Farah Ashrafzadeh, Mehran Beiraghi Toosi, Nasrin Moazen, Toktam Mohammadpoor, Reza Karami

**Affiliations:** 1 Professor of Pediatric Neurology, Ghaem Medical Center, Mashhad University of Medical Sciences, Mashhad, Iran; 2 Fellow of Pediatric Neurology, Ghaem Medical Center, Mashhad University of Medical Sciences, Mashhad, Iran; 3 Resident of Pediatrics, Ghaem Medical Center, Mashhad University of Medical Sciences, Mashhad, Iran; 4 Student of Medicine, Faculty of Medicine, Mashhad University of Medical Sciences, Mashhad, Iran

**Keywords:** Joubert syndrome, Molar tooth sign, Vermian dysgenesis

## Abstract

Joubert syndrome (JS) is a rare autosomal recessive central nervous system malformation characterized by hypoplasia of the cerebellar vermis, hypotonia and abnormal psychomotor development, along with altered respiratory pattern and various ophthalmologic features.

Here, we describe three children with Joubert syndrome in a family that had almost similar presentations, including ataxia, developmental delay, mental retardation and ocular disorders.

Prevalence of Joubert syndrome is about 1 in 100,000 live birth. It may be accompanied by other organs’ disorders. The molar tooth sign is pathognomonic for joubert syndrome that is ascertained by brain MRI.

## Introduction

Joubert syndrome (JS) is a rare autosomal recessive central nervous system malformation characterized by hypoplasia of the cerebellar vermis, hypotonia and abnormal psychomotor development, together with altered respiratory pattern and various ophthalmologic features (1). Other common characteristics of this syndrome are ataxia, oculomotor apraxia, developmental delay and mental retardation (2).

Estimates of the incidence of JS ranges between 1/80,000 and 1/100,000 live births and its recurrence risk is 25% in most families (3). Renal insufficiency is rarely reported in JS (4). Location of the affected gene of this disorder has not been definitely identified (5), so, clinical and radiological signs are necessary for diagnosis of JS. Magnetic resonance imaging (MRI) has revolutionized the diagnosis of Joubert syndrome. It revealed the midbrain malformations included fourth ventricle dilatation and superior cerebellar peduncle elongation, which forms the classically described “molar tooth sign” (6). After the first months of life, global prognosis considerably varies among JS subgroups depending on the extent and severity of organs involvement (3).

Here we describe three cases of JS in a family with mental and ocular presentations.

## Case report

We want to report three children with JS in a family with the same presentations. The only healthy child is 6 years old. She has normal IQ and development, But her tooth eruption occurred after 1 years old. Her mother’s tooth eruption had occurred in the same age.

**Case 1**

The first child of the family is a 12 years old girl, with history of developmental delay referred to Ghaem Medical Center for evaluation. Her mother recalls that the child could hold her head steady while sitting at age of one year and could sit independently at age of two years. She walked alone in age of four years. Tooth eruption occurred at age of one year. Bowel and bladder control emerged at age of four years.

She also sufferes from mental retardation, failure to thrive (FTT), strabismus, decreased visual acuity in one eye and aggressive behavior. She can put the words together but is not able to make a complete sentence and is on speech therapy.

Proximal and distal forces are normal. Deep tendon reflexes are normal too, but toe to heal test is abnormal. Her bilateral ultrasonography is normal, and has no history of any considerable respiratory disorder.

**Case 2**

She is a 10 years old girl with the same symptoms and signs as her sister, except some differences. She has developmental delay, FTT, mental retardation and decreased visual acuity in both eyes. She doesn’t have aggressive behavior.

**Figure 1:**
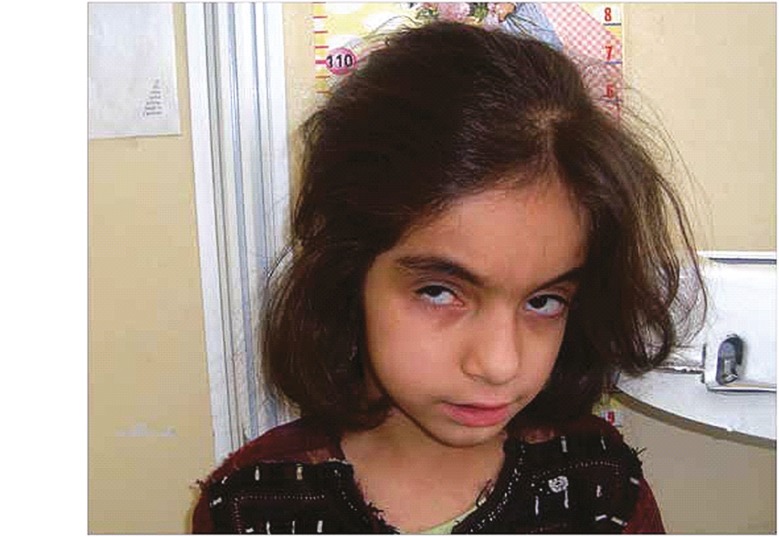
The 10 years old girl with developmental delay, FTT, mental retardation and strabismus

Her development was as same as her sister. Neurologic examinations and laboratory and imaging results were also similar (Figure 1).

**Case 3**

He is a 3.5 years old boy, brother of the aforementioned cases, with developmental delay. He could hold his head steady while sitting at age of one year and could sit independently at age of two years; he has walked without help since 1 month ago. Tooth eruption occurred at age of one year. He still has urine and stool incontinence.

**Figure 2:**
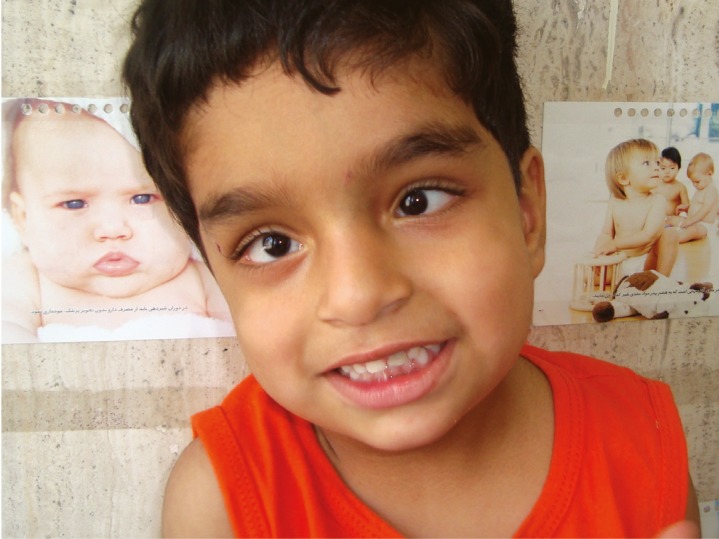
The 3.5 years old boy with bilateral abducens nerve palsy and developmental delay

He suffers from bilateral abducens nerve palsy. His limb forces and deep tendon reflexes were normal, but he had wide gait and ataxia (Figure 2).

**Figure 3:**
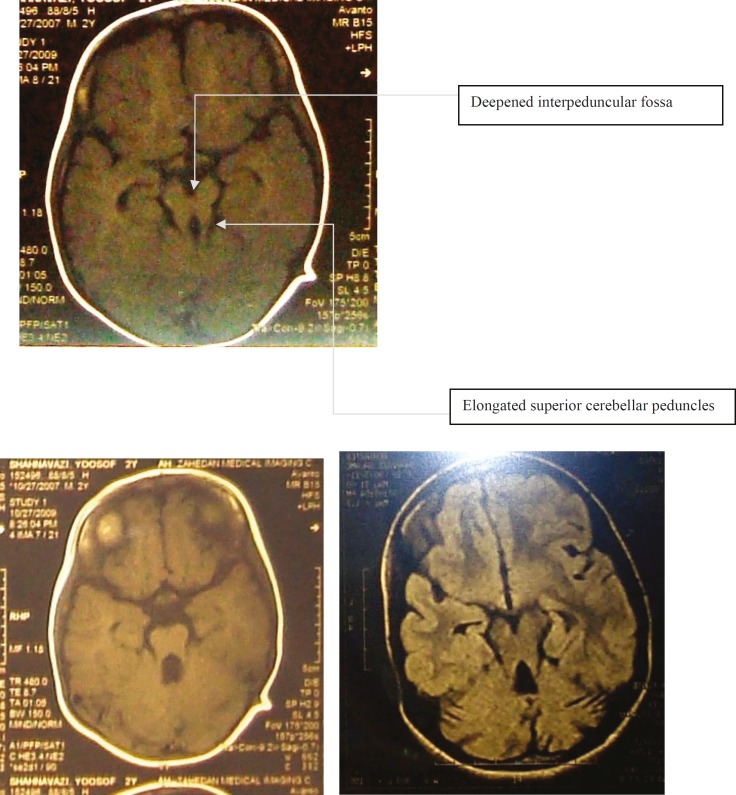
Vermian dysgenesis and horizontal superior cerebellar peduncle elongation representing the molar tooth sign pathognomonic for Joubert sy

The brain MRI without contrast shows evidences of vermian dysgenesis and superior cerebellar peduncles representing the molar tooth midbrain-hindbrain malformation which is associated with joubert syndrome (Figure 3).

Joubert syndrome is a rare (an incidence of 1/100000) disorder of central nervous system characterized by brainstem and cerebellar malformations, hypotonia, episodic hyperpnea and apnea, neuro-ophthalmologic abnormalities, and mental retardation (7).

Dr. Marie Joubert and coworkers (1969), for the first time described described four siblings with ataxia, cognitive impairment, eye movement abnormalities, cerebellar vermis agenesis, and episodic tachypnea in a French-Canadian family (8).

According to this initial report and other following reports, autosomal recessive inheritance has been deduced for JS and mutations in the eight ciliary/basal body genes, including INPP5E, AHI1, NPHP1, CEP290, TMEM67/MKS3, RPGRIP1L, ARL13B, and CC2D2A have been recognized in patients with JS. An additional locus, JBTS2 (or CORS2), was mapped via linkage analysis to chromosome 11 (9). Our cases confirm this inheritance pattern, because the parents and one child were completely healthy. Maria et al.(1997) described the midbrain-hindbrain malformation observed on MRI, as a result of hypoplasia of the midline cerebellar vermis resembling the cross section of a molar tooth (molar tooth sign’ or MTS) that is considered pathognomonic for JS (10).

The prominent problems of our cases were ataxia, developmental delay, mental retardation and ocular disorders. One of our cases had also molar tooth sign in his brain MRI, but because of their low socioeconomic level, the two others were not taken brain imaging. A diagnostic protocol to evaluate multiorgan involvement should be performed subsequent to detection of the molar tooth sign. Optimal management needs a multidisciplinary approach, with special consideration to respiratory and feeding disorders in newborns and infants. Cognitive and behavioral evaluations are recommended to provide neuropsychological support and rehabilitation services for young patients (3).

In 1997, it was reported that there is an association between JS and renal cystic disease and liver fibrosis (6), but our cases did not have any organ disorders and their ultrasonography and laboratory values were normal.

We presented three siblings of both sexes with similar clinical features, including ataxia, mental retardation, FTT, and strabismus. Their ataxia improved with age. According to their poor economic state, gene analysis couldn’t be performed and in only one of them that we could do MRI, molar tooth sign was seen that helped us to diagnose the syndrome. After one-year follow-up and rehabilitation, their abilities are much improved. We recommend that rehabilitation therapy can improve disabilities of patients with JS.
